# Decomposed Entropy and Estimation of Output Power in Deformed Microcavity Lasers

**DOI:** 10.3390/e24121737

**Published:** 2022-11-28

**Authors:** Kyu-Won Park, Kwon-Wook Son, Chang-Hyun Ju, Kabgyun Jeong

**Affiliations:** 1 Research Institute of Mathematics, Seoul National University, Seoul 08826, Republic of Korea; 2 Department of Electrical Engineering, Yeungnam University, Gyeongsan 38541, Republic of Korea; 3 School of Computational Sciences, Korea Institute for Advanced Study, Seoul 02455, Republic of Korea

**Keywords:** decomposed entropy, output power, deformed microcavity laser, directional emission

## Abstract

Park et al. showed that the Shannon entropy of the probability distribution of a single random variable for far-field profiles (FFPs) in deformed microcavity lasers can efficiently measure the directionality of deformed microcavity lasers. In this study, we instead consider two random variables of FFPs with joint probability distributions and introduce the decomposed (Shannon) entropy for the peak intensities of directional emissions. This provides a new foundation such that the decomposed entropy can estimate the degree of the output power at given FFPs without any further information.

## 1. Introduction

Deformed microcavity lasers [1,2] have been widely studied up to now owing to the existence of models for investigating various physical phenomena, such as scar [3,4], tunneling [5,6], exceptional point [7,8], and ray–wave correspondence [9,10]. They have also attracted considerable attention owing to their potential for optical applications [11]. In particular, because directional emissions are decisive for achieving high-performance lasers, various types of microcavity lasers [12,13,14,15,16,17,18] have been considered. Moreover, numerous physical systems, such as multilayer thin films [19], quantum metasurfaces [20], and nanoantennas [21] have been studied on this issue. In these studies, directional emission was only addressed in terms of a single variable. That is, the directionality and divergence angle were defined in terms of the intensity of far-field profiles (FFPs) as a function of a single variable θ [15,22,23,24]. However, these physical quantities cannot fully capture the properties of FFPs because FFPs are defined in two-dimensional microcavity lasers, rather than in one-dimensional lasers.

In particular, our previous work presented the Shannon entropy to measure directionality more efficiently [25]. However, the Shannon entropy introduced in our work was only defined by the probability distribution of a single (random) variable, as in previous studies. Accordingly, in this study, we consider the Shannon entropy, which is defined by the joint probability distribution of two random variables in a mathematically rigorous manner. In doing so, we can further introduce the subsample space associated with the peak intensities of the directional emissions. We present a new quantity called *decomposed entropy* in two-dimensional deformed microcavity lasers. This quantity behaves opposite to the previous Shannon entropy [25]. Moreover, it can effectively capture the properties of directional emission more sensitively for two different microcavity lasers. We argue that this decomposed entropy can estimate the degree of output power related to the intensities of peaks for directional emissions at given FFPs, unlike previous notions that could only estimate the directionality and divergence angle at given FFPs.

The remainder of this paper is organized as follows. In Section 2, we briefly recapitulate the probability theory in the context of mathematics and introduce the decomposed entropy. We describe our proposed system in Section 3. An analysis of the limaçon-shaped microcavity is presented in Section 4. In Section 5, the oval-shaped microcavity is analyzed. Finally, we summarize our work in Section 6.

## 2. Probability Space and Decomposed Entropy for Deformed Microcavity Lasers

### 2.1. Recapitulation of the Probability Space and Random Variables

A measurable space (S,A) with measure μ is the measure space. Here, *S* is a nonempty set, A is a sigma algebra, and μ is a map from A to a non-negative number μ: A↦[0,∞]. A measurable map *f* is a map between two measurable spaces (S,A) and $\left(\tilde{S},\tilde{A}\right)$ if, for all $a\in \tilde{A}$, the preimage of *a* under the map *f* is the element of the algebra A: $f^{-1}\left(a\right)\in A$ for all $a\in \tilde{A}$. When fulfilling μ(S)=1, the measure space (S,A,μ) corresponds to the probability space (Ω,E,P), where Ω is called a sample space, *E* is called an event space, and P is a probability function that assigns a probability to each event.

A random variable typically denoted by *X* is a measurable map from Ω to another sample space $\tilde{\Omega }$. Here, the space $\tilde{\Omega }$ typically indicates real numbers. Using these definitions, we can now define a probability mass function $\rho_{X}\left(x_{i}\right)$, where we simply use the notation $\rho (x_{i})$. The probability distribution of a discrete random variable *X* from R to [0,1] is defined by $\rho_{X}\left(x_{i}\right)=P\left(X=x_{i}\right)$ satisfying the normalization condition $\sum_{x_{i}}\rho_{X}\left(x_{i}\right)=1$. That is, $\rho_{X}\left(x_{i}\right)$ is equivalent to the probability of the occurrence of event e∈E in sample space $\Omega :P\left(X=x_{i}\right)=P\left\{e:X\left(e\right)=x_{i}\right\}$.

To perform numerical calculations for the intensities of FFPs in microcavity lasers, we consider only discrete random variables, rather than continuous ones, in our study. In addition, we should consider the two random variables X,Y to introduce the joint probability distributions for two-dimensional microcavity lasers; that is, $\rho_{X,Y}\left(x_{i},y_{j}\right)=P\left(X=x_{i},Y=y_{j}\right)$ under the normalization condition $\sum_{i,j}\rho_{X,Y}\left(x_{i},y_{j}\right)=\sum_{i,j}\rho \left(x_{i},y_{j}\right)=1$. In this case, random variables *X* and *Y* are the maps from the sample space (i.e., positions in the *X*- and *Y*-axes) to the real numbers (i.e., components of the coordinates of the *X*- and *Y*-axes). This fact imposes the probability ρ at each Cartesian coordinate $\left(X=x_{i},Y=y_{j}\right)$.

### 2.2. Decomposed Entropy of the Peak Intensities for FFPs in Two-Dimensional Microcavity Lasers

Shannon’s information entropy is a relevant measure of the average information content or can be interpreted as the complexity of a given probability distribution of a random variable. It was first developed by Claude Shannon in his seminal paper on “communication theory” [26] and has been extensively exploited in numerous areas. The Shannon entropy has been employed for variability, for example, in molecular descriptors [27] and protein sequences [28]. This has also been exploited in economics [29] and market efficiency [30].

The discrete Shannon entropy of probability distributions ${\left\{\rho \left(x_{i}\right)\right\}}_{i=1}^{N}$ for the random variable *X* is formally defined as

(1) $$\begin{array}{c} H=-\sum_{i=1}^{N}\rho \left(x_{i}\right)\log\rho \left(x_{i}\right) \end{array}$$

under the normalization condition $\sum_{i=1}^{N}\rho \left(x_{i}\right)=1$.

First, to handle the Shannon entropy of peak emissions for directional emissions, we assume that the total sample space Ω can be decomposed into subsample space $\bar{\Omega }$ and its complement ${\bar{\Omega }}^{c}$: in other words, $\Omega =\bar{\Omega }\cup {\bar{\Omega }}^{c}$ under the condition $\{\rho \left(x_{1}\right)$, …, $\rho \left(x_{j}\right)\}\in \bar{\Omega }$ and $\{\rho \left(x_{j+1}\right)$, …, $\rho \left(x_{N}\right)\}\in {\bar{\Omega }}^{c}$, resulting in $\sum_{i=1}^{j}\rho \left(x_{i}\right)+\sum_{i=j+1}^{N}\rho \left(x_{i}\right)=1$. Under these conditions, we can assume that the Shannon entropy can be decomposed into two parts in analogy to the Boltzmann–Gibbs entropy [31,32] as follows:

(2) $$\begin{array}{c} H_{T}=\bar{H}+{\bar{H}}^{c}. \end{array}$$

Here, $\bar{H}=-\sum_{i=1}^{j}\rho \left(x_{i}\right)\log\rho \left(x_{i}\right)$ is defined only in $\bar{\Omega }$, and ${\bar{H}}^{c}=-\sum_{i=j+1}^{N}\rho \left(x_{i=j+1}\right)$$\log\rho (x_{i=j+1})$ is defined solely in ${\bar{\Omega }}^{c}$. Employing these conventions, we analyze new properties of the decomposed entropy on two shapes (i.e., limaçon- and oval-shaped) of microcavity lasers.

## 3. Illustration of the Proposed Microcavity Lasers

In our previous paper, we considered the limaçon-shaped microcavity and oval-shaped microcavity lasers as candidates for directional emissions. The geometrical boundary of the limaçon-shaped microcavity is defined as R(θ)=(1+χcosθ), and that of the oval-shaped microcavity laser is defined as $x^{2}/a^{2}+\left(1+\varepsilon x\right)y^{2}/b^{2}=1$. For the limaçon-shaped microcavity, χ is the deformation parameter in the range of 0.43≤χ≤0.478, and θ is the angle in polar coordinates. For the oval-shaped microcavity laser, parameters *a* and *b* are the major and minor axes of an ellipse with fixed values a=1.0 and b=1.03, and ε is the deformation parameter in the range of 0.043≤ε≤0.058. Both microcavity lasers have the same effective refractive index n=3.3 for InGaAsP semiconductors.

**Figure 1:**
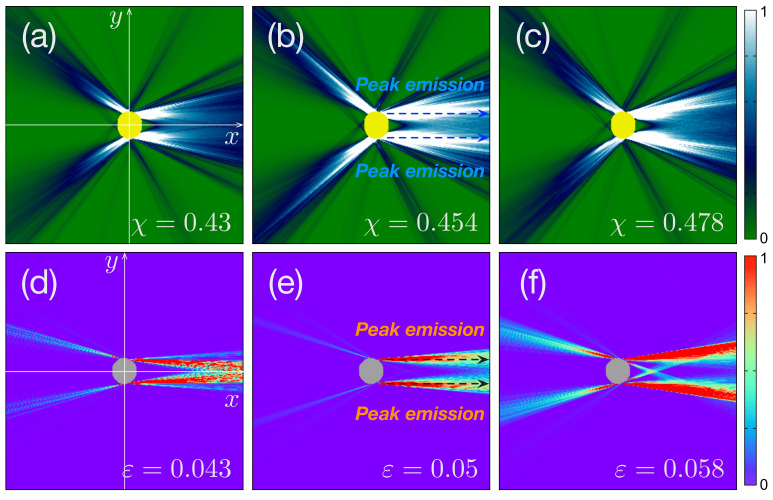
Some of the representative far-field profiles (FFPs) in a limaçon-shaped cavity and oval-shaped microcavity lasers [25]. Figures (**a**–**c**) are the FFPs of a limaçon-shaped cavity in the Cartesian coordinate within −10≤x≤10 and −10≤y≤10 at each deformation χ=0.43, χ=0.454, and χ=0.478, respectively. Figures (**d**–**f**) are the FFPs of an oval-shaped cavity in the Cartesian coordinate within −10≤x≤10 and −10≤y≤10 at each deformation ε=0.043, ε=0.05, and ε=0.058, respectively. The thick-dotted lines in Figures (**b**,**e**) depict two peak emissions of the deformed microcavity lasers.

Figure 1a–c display some profiles of the representative intensities of FFPs at each deformation χ=0.43, χ=0.454, and χ=0.478 for the limaçon-shaped microcavity, respectively. For the case of an oval-shaped microcavity, Figure 1d–f display some of the representative intensities of FFPs at each deformation ε=0.043, ε=0.05, and ε=0.058, respectively. The intensities were calculated from the transmitted rays using the Fresnel equations. To reproduce the previous results, we again considered transverse magnetic (TM) modes for a limaçon-shaped microcavity and transverse electric (TE) modes for an oval-shaped microcavity.

In addition, to perform numerical calculations for the ray simulations, we considered the FFPs in the Cartesian coordinates within −10≤x≤10 and −10≤y≤10 for numerical convenience. That is, we discretized the (*x*, *y*)-coordinates in the range of x∈[−10,10] and y∈[−10,10] into a 2000×2000 grid. Additionally, the thick dotted lines in Figure 1b,e depict the peak intensities of directional emissions.

## 4. Analysis on a Limaçon-Shaped Microcavity

### 4.1. Marginal and Conditional Intensities in a Limaçon-Shaped Microcavity

**Figure 2:**
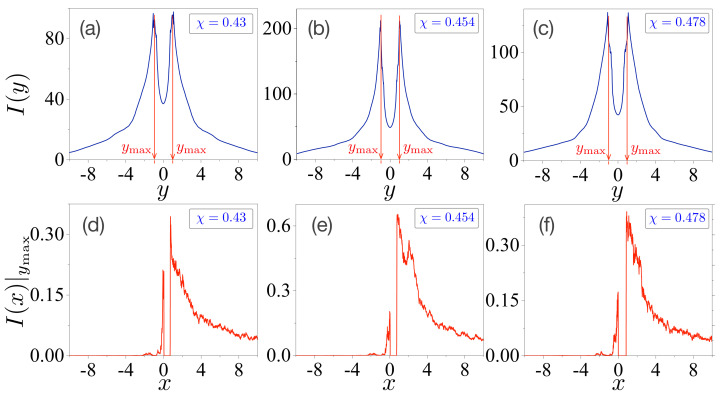
Marginal and conditional intensities in a limaçon-shaped microcavity laser. Plots (**a**–**c**) are marginal intensities as a function of the *y*-coordinate at each deformation χ=0.43, χ=0.454, and χ=0.478, respectively. They have maximal values around y=±0.99 denoted by $y_{\max}$. Plots (**d**–**f**) are conditional intensities as a function of the *x*-coordinate at the fixed value at $Y=y_{\max}$.

To address the peak emissions at the given FFPs in the Cartesian coordinate system, we first need to specify the locations of the *y*-coordinate for peak emissions because the directional light propagates along the *x*-axis at a specific *y*-coordinate. For this approach, we introduce the marginal intensity, which is defined as

(3) $$\begin{array}{c} I\left(y\right)=\sum_{x_{i}=1}^{N}I\left(x_{i},y_{j}\right) \end{array}$$

with N=2000. Plots (a–c) in the upper panels of Figure 2 present the results for each deformation parameter χ=0.43, χ=0.454, and χ=0.478. We observed that the values of I(y) have maximal values around y=±0.99 denoted by $y_{\max}$. Thus, we can also propose the quantity of the conditional intensity as a function of the *x*-coordinate ${I\left(x\right)|}_{y_{\max}}$ at the fixed value of $y_{\max}≃\pm 0.99$ to specify the FFPs for the peak emissions. The results are plotted in Figures (d–f) in the lower panels of Figure 2. These plots can be interpreted as the peak intensities of directional light propagation in the microcavity lasers. Note that the overall values of ${I\left(x\right)|}_{y_{\max}}$ at χ=0.454 are larger than those at χ=0.43 and χ=0.478. It is also expected that the relative portion ${I\left(x\geq 0\right)|}_{y_{\max}}{/I\left(x\leq 0\right)|}_{y_{\max}}$ at χ=0.454 be larger than those at χ=0.43 and χ=0.478. Accordingly, this directly indicates that the forward emissions of FFPs at χ=0.454 are larger than those of the others.

### 4.2. Total and Decomposed Entropies in a Limaçon-Shaped Microcavity

Directional emissions have been investigated in terms of directionality and divergence angle at given FFPs so far [21,22,23,24,25]. In this subsection, we present a method to estimate the degree of output power at given FFPs by exploiting the decomposed Shannon entropy. Accordingly, we should first have probability distributions of the given random variables associated with the FFPs. As mentioned in Section 2.2 of Section 2, we consider the joint probability distributions ρ(x,y) of the two random variables X,Y by normalizing the intensity I(x,y) of the FFPs under the condition $\sum_{i=1}^{N}\sum_{j=1}^{N}I\left(x_{i},y_{j}\right)=1$ with N=2000. In this case, the total sample space Ω comprises a 2000×2000 grid for the discretized (*x*,*y*)-coordinates in the range of x∈[−10,10] and y∈[−10,10].

**Figure 3:**
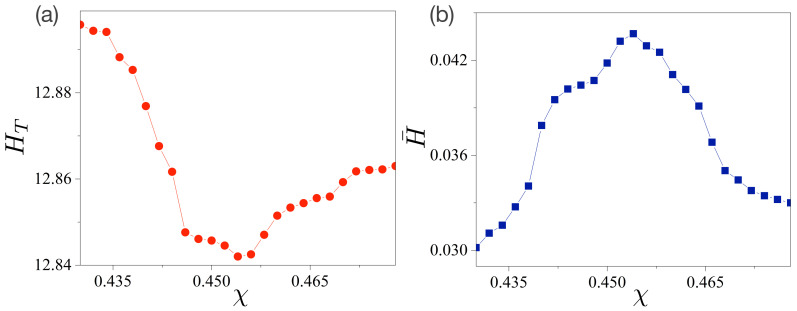
Total and decomposed entropies in a limaçon-shaped microcavity laser. (**a**) Total entropy $H_{T}$ with joint probability ρ(x,y) depending on the deformation parameter χ. $H_{T}$ has a minimal value at χ=0.454. (**b**) Decomposed entropy $\bar{H}$ of the peak intensities of FFPs. $\bar{H}$ has a maximal value at χ=0.454.

Let us consider the total sample space Ω related to the FFPs and analyze it using the total entropy $H_{T}$, which is defined as follows:

(4) $$\begin{array}{c} H_{T}=-\sum_{i=1}^{N}\sum_{j=1}^{N}\rho \left(x_{i},y_{j}\right)\log\rho \left(x_{i},y_{j}\right), \end{array}$$

with N=2000. As shown in Figure 3a, the value of $H_{T}$ is minimal at χ=0.454, and its overall behavior is similar to the Shannon entropy of a single random variable angle θ. (See details in our previous paper [25].) This result indicates that the delocalization or complexity of the intensity distributions of the FFPs in the (x,y)-coordinates are minimized at χ=0.454. Equivalently, the directionality of FFPs is maximized at χ=0.454.

Now, we decompose the total sample space Ω into subsample space $\bar{\Omega }$ and its complement ${\bar{\Omega }}^{c}$. Because defining the subsample space $\bar{\Omega }$ has no restrictions, we can select the normalized peak intensities of the directional emissions as the elements of $\bar{\Omega }$, which are depicted as dotted arrows in Figure 1b,e, i.e., $\left\{P\left(X\geq 0,Y=y_{\max}\right)\right\}\in \bar{\Omega }$. Note that we chose only X≥0 to handle forward emissions solely. Thus, the decomposed entropy $\bar{H}$ is defined as follows:

(5) $$\begin{array}{c} \bar{H}=-\sum_{i=1}^{N/2}\rho \left(x_{i},y_{\max}\right)\log\rho \left(x_{i},y_{\max}\right). \end{array}$$

The explicit calculations are presented in Figure 3b. Evidently, its absolute values are significantly smaller than those of $H_{T}$ in Figure 3a. Most importantly, however, it should be noted that its overall pattern is the opposite of that of the total entropy $H_{T}$ in Figure 3a. The value of $\bar{H}$ is maximized at χ=0.454. Therefore, we conjecture that $\bar{H}$ can estimate the output power because $\bar{H}$ is related to the spreading (or delocalization) of the intensity of the directional light propagation embedded in the FFPs.

## 5. Analysis on an Oval-Shaped Microcavity

### 5.1. Marginal and Conditional Intensities in an Oval-Shaped Microcavity

**Figure 4:**
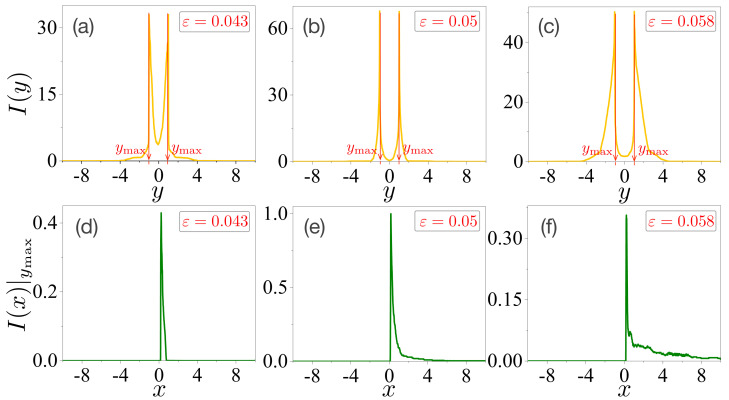
Marginal and conditional intensities in the oval-shaped microcavity laser. Plots (**a**–**c**) are marginal intensities as a function of the *y*-coordinate at each deformation ε=0.043, ε=0.05, and ε=0.058, respectively. They have maximal values around y=±1.01, as denoted by $y_{\max}$. Plots (**d**–**f**) are conditional intensities as a function of the *x*-coordinate at the fixed value of $Y=y_{\max}$.

To validate the generality of our argument, we investigated the decomposed entropy of an oval-shaped microcavity laser. The marginal and conditional intensities of the FFPs in the oval-shaped microcavity laser were obtained in the same manner as in Section 4.1 in Section 4, and Figure 4 shows the results.

Figure 4a–c present the marginal intensities as a function of the *y*-coordinate at each deformation χ=0.043, χ=0.05, and χ=0.058, respectively. The values of I(y) have maximum values around y=±1.01, as denoted by $y_{\max}$. The conditional intensities as a function of the *x*-coordinate at a fixed value of $Y=y_{\max}$ are plotted in Figure 4d–f. We also expect that the relative portions ${I\left(x\geq 0\right)|}_{y_{\max}}{/I\left(x\leq 0\right)|}_{y_{\max}}$ in Figure 4d–f to be larger than those shown in Figure 2d–f. This can reveal that the forward emissions of the FFPs in the oval-shaped microcavity are larger than those in the limaçon-shaped microcavity.

### 5.2. Total and Decomposed Entropies in the Oval-Shaped Microcavity

**Figure 5:**
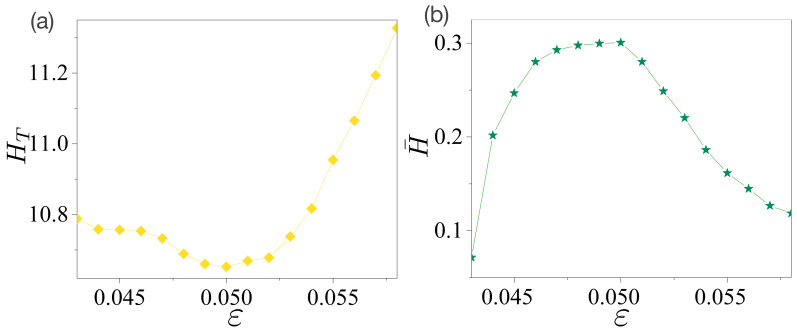
Total and decomposed entropies in an oval-shaped cavity. (**a**) Total entropy $H_{T}$ with joint probability ρ(x,y) depending on the deformation parameter ε. $H_{T}$ has a minimal value at ε=0.050. (**b**) Decomposed entropy $\bar{H}$ of the peak intensities of the FFPs. The entropy $\bar{H}$ has a maximal value at ε=0.050.

The total entropy $H_{T}$ and decomposed entropy $\bar{H}$ were also obtained in the same manner as in Section 4. The results are presented in Figure 5. The value of $H_{T}$ in Figure 5a is minimized at χ=0.05, and its overall values are smaller than those in Figure 3a. These results agree with our previous results [25]. However, unlike the case of the limaçon-shaped microcavity, its overall behavior exhibits a slight difference from that of the Shannon entropy of a single random variable angle θ. The value of $H_{T}$ at ε=0.058 is larger than that of $H_{T}$ at ε=0.043, whereas the Shannon entropy in ref. [25] shows the opposite behavior. This can be attributed to the fact that the primary emission route of the FFPs in the limaçon-shaped microcavity hardly changes depending on the parameter χ, whereas that of the FFPs in the oval-shaped microcavity changes depending on ε. That is, the FFPs in the oval-shaped microcavity had four primary emission routes at ε=0.043, whereas they had six primary emission routes at ε=0.058. Therefore, the total Shannon entropy of the probability distributions of a single random variable θ cannot capture the total information of the FFPs, whereas those of the two random variables (X,Y) can.

The decomposed entropy $\bar{H}$ of the peak intensities of the directional emissions is shown in Figure 5b. It is maximized at χ=0.05 with a value of $\bar{H}≃0.3$. Thus, we can suggest that the main results are as follows. The degree of directional emission defined by the intensity of angle I(θ) shows a similar order of magnitude for the two different microcavity lasers, that is, the directionality defined by $U_{W}$, $U_{C}$, or $S_{N}$, and the divergence angle exhibits a similar order of magnitude in both microcavity lasers as in ref. [25]. However, the decomposed entropy $\bar{H}$ in Figure 5b is almost ten times larger than that of the decomposed entropy $\bar{H}$ in Figure 3b. Accordingly, the decomposed entropy $\bar{H}$ can more sensitively capture the properties of directional emissions for different microcavity lasers. Here, we also conjecture that the decomposed entropy $\bar{H}$ can estimate the output power at the given FFPs without any further information.

## 6. Conclusions

We introduced the total entropy and decomposed entropy for the joint probabilities of two random variables (X,Y) and examined the possibility of estimating the output power of deformed microcavity lasers at given FFPs. The total entropy $H_{T}$ was defined in the total sample space Ω, whereas the decomposition entropy $\bar{H}$ was defined in the subsample space $\bar{\Omega }$, which consisted of normalized peak intensities of the directional emissions. The total entropy $H_{T}$ behaved roughly similar to the Shannon entropy for a single random variable θ. On the contrary, the decomposed entropy $\bar{H}$ behaved in an opposite way to $H_{T}$. The decomposed entropy $\bar{H}$ in the oval-shaped microcavity was ten times larger than the decomposed entropy $\bar{H}$ in the limaçon-shaped microcavity. Thus, $\bar{H}$ could more efficiently detect the properties of directional emission for different microcavity lasers than the directionality and divergence angles. It could also estimate the degree of the output power at given FFPs without any further information. The output power plays a crucial role in designing microcavity lasers or other physical systems as quality factor and directional emission do [33,34,35]. In addition, because our notion depends only on the given FFPs, it can be applied to any type of microcavity laser or antenna system. Moreover, unlike conventional methods, our method can be applied to three-dimensional physical systems when three random variables correspond to the appropriate coordinates of the $R^{3}$ space. Accordingly, we hope that our results can help in designing high-performance lasers or other physical systems concerning directional emissions.

Finally, it is worth mentioning that the decomposed entropy is quite different from the conditional entropy, more precisely, the entropy of *X* conditioned on *Y* taking the value $Y=y_{\max}$, i.e., $H(X|Y=y_{\max})$. In this case, the total sample space Ω collapses into the subsample space $\bar{\Omega }$ and loses information about its complement ${\bar{\Omega }}^{c}$.

## Data Availability

Not applicable.
